# Double slope solar still distillate output data set for conventional still and still with or without reflectors and PCM using high TDS water samples

**DOI:** 10.1016/j.dib.2019.103852

**Published:** 2019-03-20

**Authors:** Siddharaj V. Kumbhar

**Affiliations:** Consultant Engineer, HVACR & Solar Systems, India

**Keywords:** Solar still, Efficiency, Distillate output, TDS, PCM, Reflectors

## Abstract

The rate of distillate and temperatures are important factors for analyzing the solar stills. The higher rate of distillate and hence the higher efficiency can be achieved by engrossing different measures to develop the still, for example; double slope instead of single slope, incorporating reflectors for maximum insolation, use of PCM for increasing the time period of operation of solar still after sun hours. The data associated with developed double slope solar still is presented to correlate variation of distillate output with the still basin and glass cover temperatures as well as stills with and without reflectors and PCM for different types of high TDS level water samples. The data set of temperature and distillate output can be used to analyze the working of stills for its efficiency and effectiveness in terms of distillate yield.

**Table:** Specifications table

Subject area	*Solar Energy & Desalination*
More specific subject area	*Solar Distillation*
Type of data	*Table, graph, figure*
How data was acquired	*Temperature measurements, flow measurements,* pH *and TDS tests*
Data format	*Raw, analyzed*
Experimental factors	*High TDS Water Samples* *Sample 1-brackish water (high contents of Sodium Carbonates – 40% soap solution)* *Sample 2-* waste water of RO plant (45% TDS)
Experimental features	*The temperatures measured by using 12 Channel DTI-101 M with J-type thermocouples.* *Output flow measurements done using* 1000ml *measuring cylinder of model237O2.* pH *measured using PHH222* pH *meter.* *TDS measurement done by PHH-126 m.*
Data source location	*Solapur, Maharashtra, INDIA*
Data accessibility	*The data is with this article*
Related research article	Kumbhar, S. V., Status of Groundwater Contamination and Health Hazards Due to Arsenic and Fluorides: A Review. *International Journal of Water Resources*, 2017, Vol. 3, Issue 1, pp. 1–4 [1].

**Table:** 

**Value of the data** • The data can be used to correlate distillate output with respect to still basin temperature and time • The data demonstrates greater insights on how distillate output varies with glass cover temperatures • The extensive comparison of conventional solar still and developed solar still (comprising of double slope, reflectors, PCM) shall help for obtaining higher distillate output • Use of high TDS water in basin facilitates in determination of effectiveness in working of still as to similar purification techniques

## 1 Data

The experimental investigations performed on the developed solar still acquires the temperatures of basin, water and glass cover and the corresponding distillate output [Table 1, Table 2, Table 3, Table 4, Table 5, Table 6]. The values are plotted with respect to time and temperature [Fig. 1, Fig. 3, Fig. 5, Fig. 7, Fig. 9, Fig. 11]. The distillate output is plotted with respect to time [Fig. 2, Fig. 4, Fig. 6, Fig. 8, Fig. 10, Fig. 12].

**Table 1:** Observations case-1.

Time (hours)	Temperature of Basin water (°C)	Temperature of Basin (°C)	Glass Cover Temperature (°C)		Distillate output (ml)
			Inside	Outside	
10	25	31	36	34	0
11	55	55	48	42	110
12	68	60	55	45	260
13	71	68	60	47	530
14	65	64	59	48	750
15	68	72	55	44	940
16	60	67	50	42	1070

**Table 2:** Observations case-2.

Time (hours)	Temperature of Basin water (°C)	Temperature of Basin (°C)	Glass Cover Temperature (°C)		Distillate output (ml)
			Inside	Outside	
10	30	32	36	32	0
11	53	56	50	39	110
12	58	60	57	41	270
13	55	58	56	46	520
14	56	57	55	41	770
15	56	57	48	40	985
16	52	53	47	39	1135

**Table 3:** Observations case-3.

Time (hours)	Temperature of Basin water (°C)	Temperature of Basin (°C)	Glass Cover Temperature (°C)		Distillate output (ml)
			Inside	Outside	
10	30	32	30	32	0
11	61	64	57	36	56
12	68	70	69	47	371
13	68	72	61	46	610
14	69	74	64	42	859
15	61	63	60	42	1108
16	59	60	59	40	1260

**Table 4:** Observations case-4.

Time (hours)	Temperature of Basin water (°C)	Temperature of Basin (°C)	Glass Cover Temperature (°C)		Distillate output (ml)
			Inside	Outside	
10	30	31	30	33	0
11	58	62	55	37	100
12	65	69	67	45	330
13	74	74	68	46	615
14	69	70	61	44	880
15	62	64	59	43	1105
16	60	61	58	42	1255

**Table 5:** Observations case-5.

Time (hours)	Temperature of Basin water (°C)	Temperature of Basin (°C)	Glass Cover Temperature (°C)		Distillate output (ml)	Wax Temperature (°C)
			Inside	Outside		
10.30	30	32	31	32	0	32
11.30	62	64	57	36	160	57
12.30	72	74	68	47	460	71
13.30	67	70	62	43	780	74
14.30	60	65	54	42	920	77
15.30	62	59	54	39	1130	60
16.30	61	65	53	42	1350	60
17.30	55	63	48	38	1480	59
18.30	51	52	40	37	1540	54
19.30	48	50	35	34	1580	52

**Table 6:** Observations case-6.

Time (hours)	Temperature of Basin water (°C)	Temperature of Basin (°C)	Glass Cover Temperature (°C)		Distillate output (ml)	Wax Temperature (°C)
			Inside	Outside		
10.30	32	33	30	32	0	32
11.30	63	64	58	35	165	58
12.30	71	73	68	45	482	72
13.30	73	71	63	44	800	74
14.30	65	68	58	42	980	77
15.30	62	64	54	39	1120	76
16.30	65	70	56	42	1365	61
17.30	55	64	50	41	1490	60
18.30	54	52	40	35	1560	59
19.30	51	50	38	34	1610	55

**Fig. 1:**
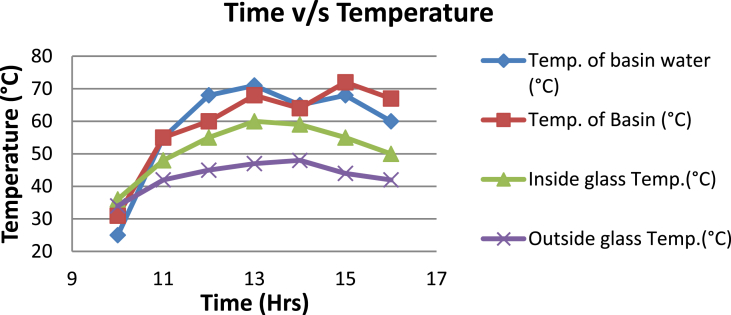
Graph of Time v/s Temperature (Case-1).

**Fig. 2:**
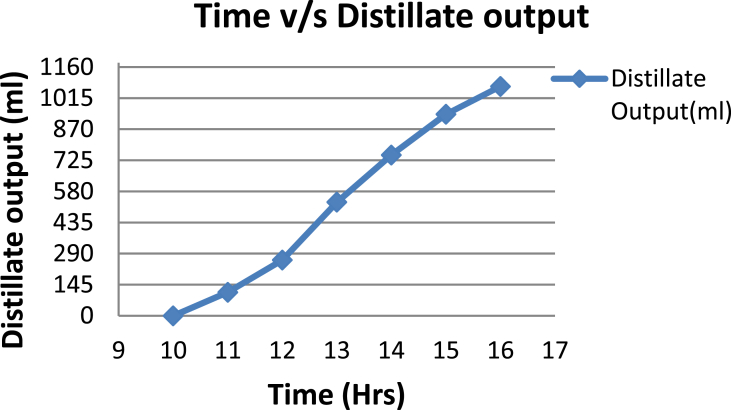
Graph of Time v/s Distillate Output (Case-1).

**Fig. 3:**
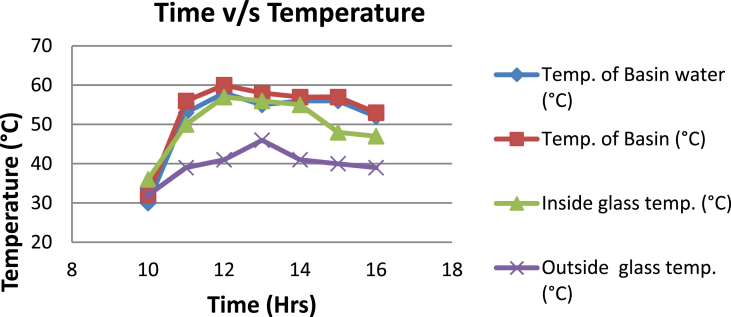
Graph of Time v/s Temperature (Case-2).

**Fig. 4:**
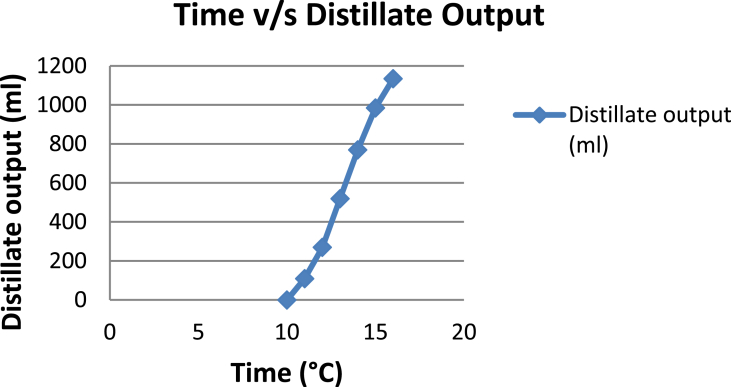
Graph of Time v/s Distillate Output (Case-2).

**Fig. 5:**
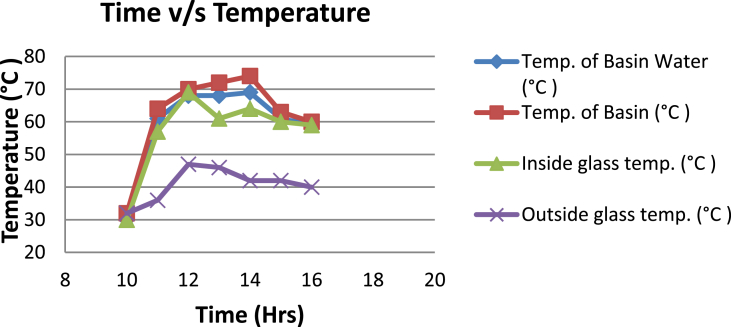
Graph of Time v/s Temperature (Case-3).

**Fig. 6:**
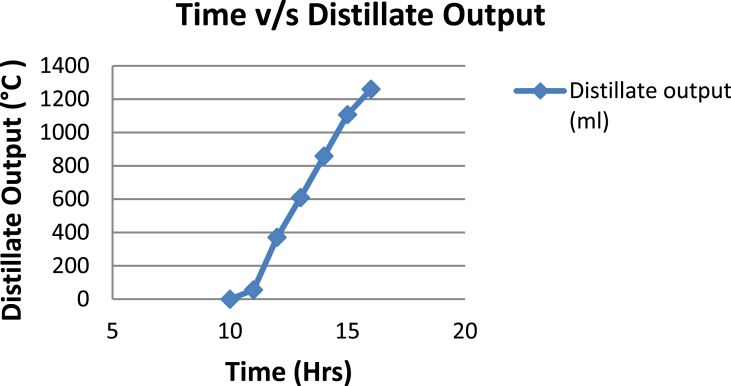
Graph of Time v/s Distillate Output (Case-3).

**Fig. 7:**
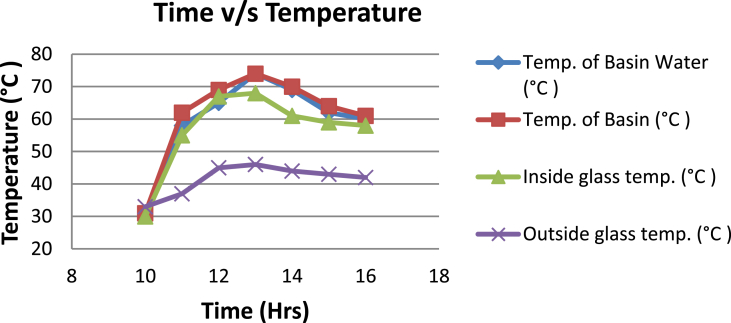
Graph of Time v/s Temperature (Case-4).

**Fig. 8:**
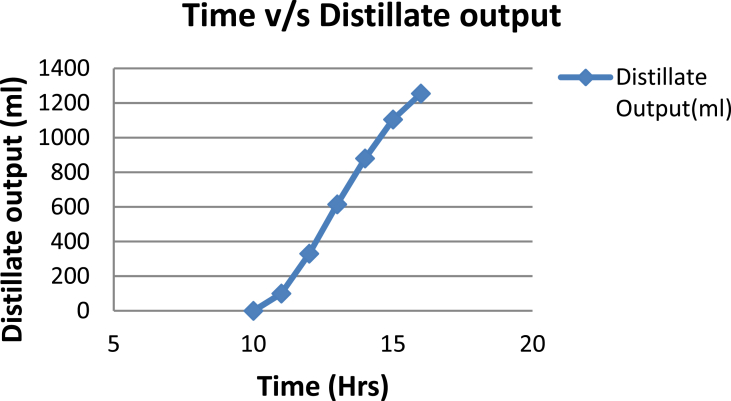
Graph of Time v/s Distillate Output (Case-4).

**Fig. 9:**
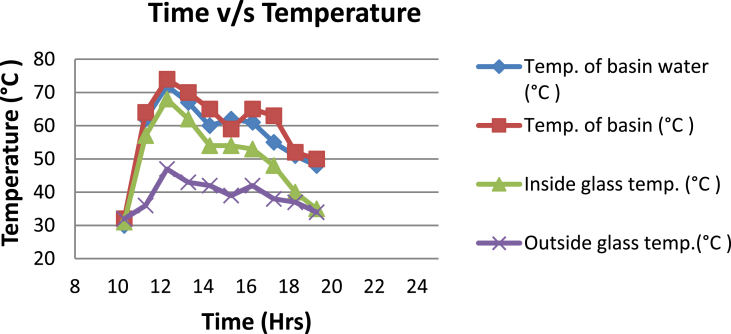
Graph of Time v/s Temperature (Case-5).

**Fig. 10:**
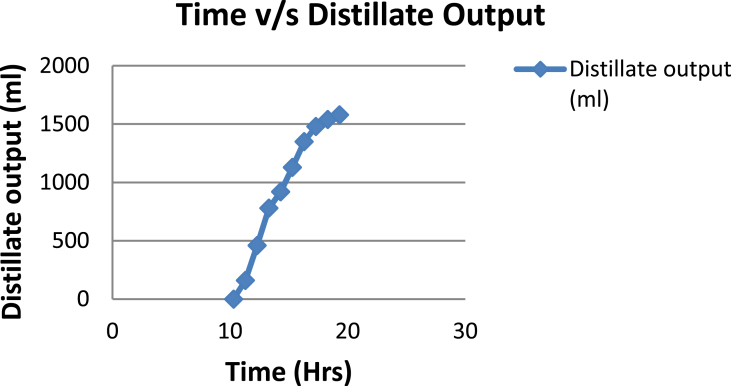
Graph of Time v/s Distillate Output (Case-5).

**Fig. 11:**
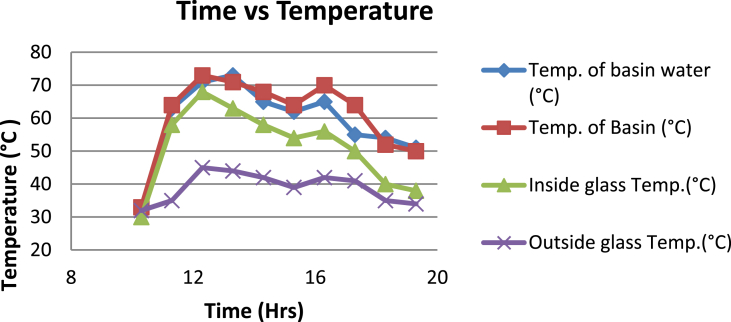
Graph of Time v/s Temperature (Case-6).

**Fig. 12:**
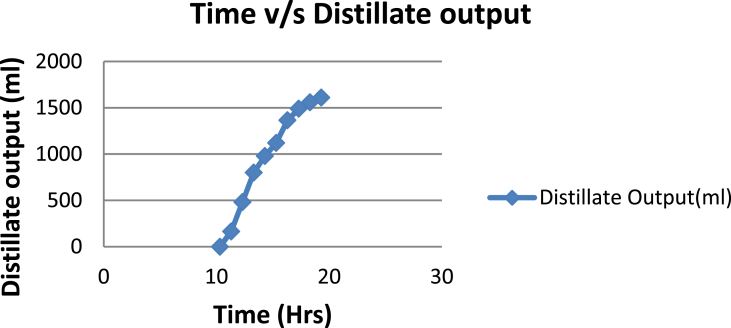
Graph of Time v/s Distillate output (Case-6).

The samples of water used are:

Sample 1 – Brackish Water (High contents of Sodium Carbonates – 40% soap solution).

Sample 2 – Waste water of RO plant (45% TDS).

The experiments are conducted on solar still with/without reflectors and/or PCM.

The case by case basis experimental investigations are then done with different combinations as follows.

Case-1: Still – Without reflector and without PCM tank; Sample 1.

Case-2: Still – Without reflector and without PCM tank; Sample 2.

Case-3: Still – With reflector and without PCM; Sample 1.

Case-4: Still – With reflector and without PCM; Sample 2.

Case-5: Still – With reflector and with PCM; Sample 1.

Case-6: Still – With reflector and with PCM; Sample 2.

Fig. 1, Fig. 3, Fig. 5, Fig. 7, Fig. 9, Fig. 11 shows the typical variation of temperature with time throughout the sun hours. It is seen that temperature reaches peak value of 70–75 °C around 1200–1400 Hrs of day time, on clear sunny day. While the distillate output rate is high around 1300–1600 Hrs, as shown in Fig. 2, Fig. 4, Fig. 6, Fig. 8, Fig. 10, Fig. 12, which resembles heat gain by water and time taken for evaporation and condensation.

Different water mixtures with variable TDS levels may have different distillate output depending on water level in basin and ratio of TDS to water. Water mixtures of high specific heat and thermal conductivity TDS, add to requirement of more heat to evaporate water thus affecting efficiency.

### 1.1 Test reports of distilled water

Analysis of Sample 1 – Brackish Water (High contents of Sodium Carbonates – 40% soap solution) [Table 7]

**Table 7:** Distilled water analysis (sample 1).

Sr. No.	Test	Brackish Water Input	Data of Distillate Water Output	Acceptable limit
1	pH	8.3	7.1	6.5–8.5
2	Alkalinity(mg/l)	172	120	200
3	Hardness (mg/l)	236	160	200
4	Chlorides(mg/l)	278	206	250
5	Turbidity (NTU)	3.8	0.1	1
6	Total Dissolved Solids (mg/l)	780	250	500

Analysis of Sample 2 – Waste water of RO plant (45% TDS)[Table 8]

**Table 8:** Distilled water analysis (sample 2).

Sr. No.	Test	RO plant waste water Input	Data of Distillate Water Output	Acceptable limit
1	pH	7.82	7.3	6.5–8.5
2	Alkalinity(mg/l)	166	168	200
3	Hardness(mg/l)	178	166	200
4	Chlorides(mg/l)	235	228	250
5	Turbidity (NTU)	1.1	0.6	1
6	Total Dissolved Solids (mg/l)	820	260	500

**Table 9:** Efficiency of solar still for different combinations (for case 1–6).

Case	Efficiency %
1	27.98
2	29.38
3	32.95
4	32.69
5	41.31
6	42.10

## 2 Experimental design, materials, and methods

**Fig. 13:**
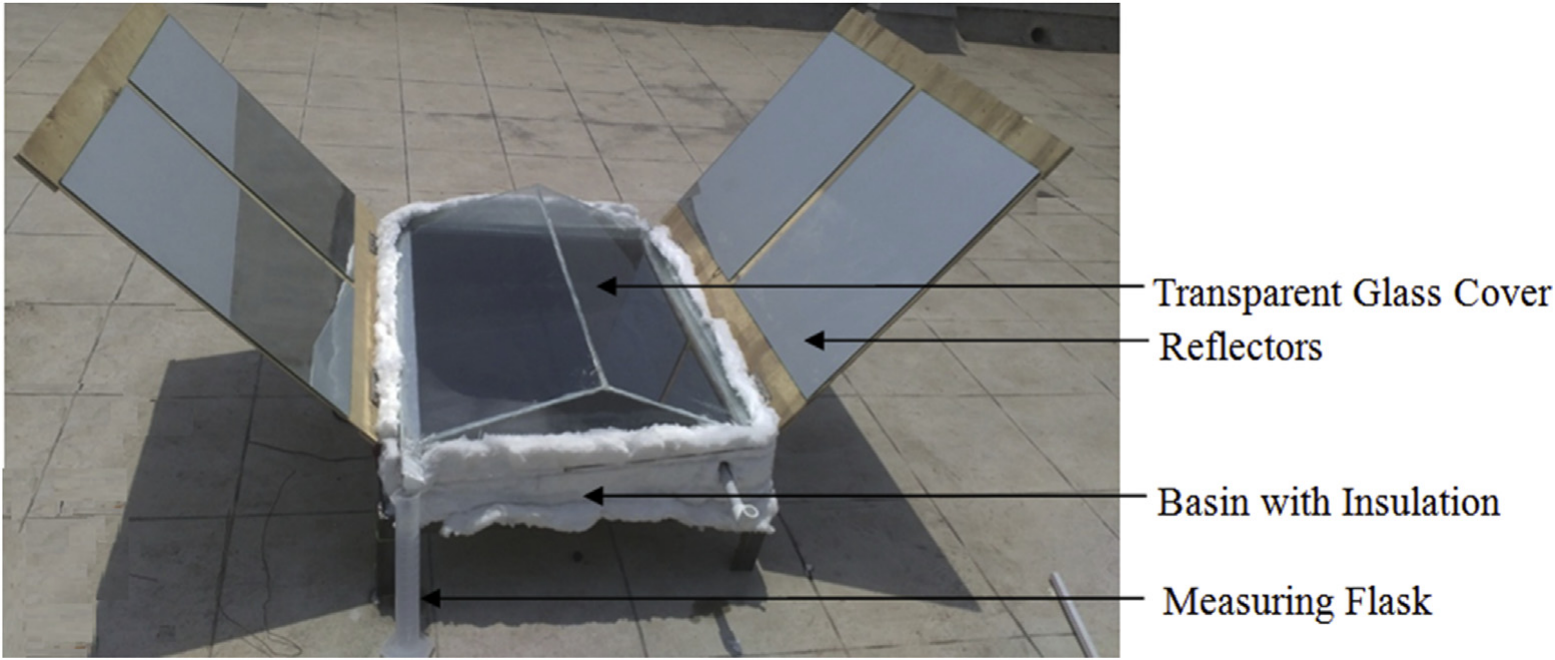
Actual setup of double slope solar still.

The design methodology of the project involves designing symmetrical double slope solar still as per the output desired. The solar still is designed for an estimated output of around 2 liters. In addition, external reflectors and PCM energy storage unit has been used to increase the output efficiency [1], [2]. Fig. 13 shows the actual setup of Double Slope Solar Still.

Base Area:

Estimation of base area is required for determining the quantum of incident solar radiation necessary for producing the desired output. With a desired output of 2 liters of water, the amount of solar energy required can be calculated as

(4) $$M_{w}\;=\;\frac{Q_{req}}{Latent\;Heat\;of\;vaporiazation}$$

Therefore, $Q_{req}=4520\;kJ$

Now, In order to calculate the amount of incident solar energy ($Q_{incident})$ it is needed to analyze the data of the average amount of solar energy incident in Solapur every month. The average solar energy received in Solapur throughout the year is $533\;W/m^{2}$

I = Average solar incident radiation for Solapur region.

I = $533\;W/m^{2}$

Generally glass transmits 80% of light through it. Let us consider 70% of solar rays incident are utilized.

(5) $$Q_{available}=\tau_{glass}\times I$$

$$Q_{available}=331.8\;W/m^{2}$$

$$Thus,Q_{incident}=Q_{req}$$

Therefore area required to get 4520 kJ is

(6) $$\frac{Q_{req}}{Q_{available}}$$

Assuming that the system works 6 hrs/day

$$Q_{available}=7166.88\;kJ/m^{2}$$

$$Area\;required=0.63\;m^{2}$$

Reflector:

In order to increase the incident solar radiation into the still, the external reflectors are incorporated [1], [2]. Due to this evaporation rate is increased. The additional amount of solar energy incident on the solar still, due to the external reflector can be calculated as follows.

Considering 4000 KJ of excess energy is available, area of reflector required to obtain this energy.

Average solar incident radiation for Solapur region is 533 $W/m^{2}$

(7) $$Q_{req}=\tau_{glass}\times A_{reflector}\times I$$

$$Q_{req}=331.8\;\;W$$

Phase Change Material (PCM):

For 1 kg pure water, additional 2600kJ energy is needed. To store 2600 kJ energy in storage material i. e paraffin wax,

(8) $$Q={\left\{m\times C_{p}\left(T_{melting}-T_{ambient}\right)\right\}}_{solid}+\left(m\times L_{h}\right)+\left\{m\times C_{p}\left(T_{max}-T_{melting}\right)\right\}$$

Therefore, mass of PCM, m=6.5kg

Dimensions of Solar Still [Table 10]:

**Table 10:** Dimensions of solar still.

Sr. No.	Parameters	Values
1	Area of basin	0.65 $m^{2}$
2	Height of basin	0.15 m
3	Area of glass	0.648$m^{2}$
4	Thickness of glass	0.004 m
5	Inclination of glass	23°

### 2.1 Materials and methods

#### 2.1.1 Basin (Fig. 14)

Shape = Rectangular

**Fig. 14:**
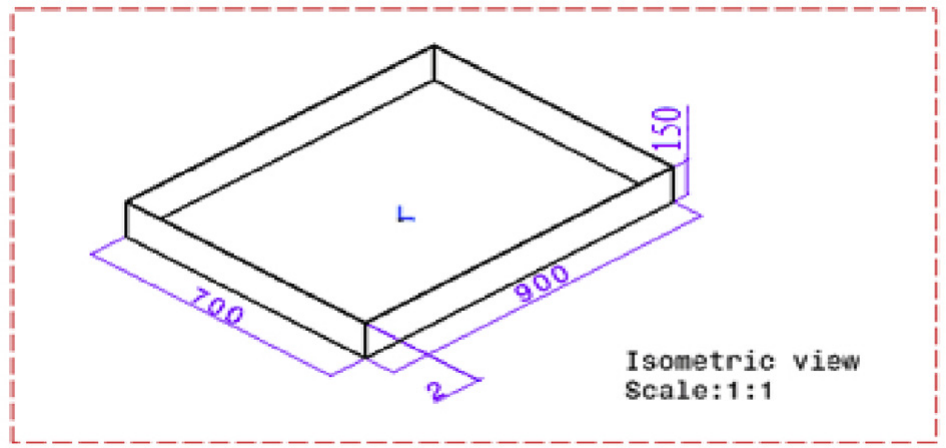
CAD model of basin.

Area = 0.9 m ×0.7 m

Height = 0.15 m

Material = G.I

Specific heat of G.I. = 0.45 KJ/kg.K

Black spray painted base, so as to increase the absorptivity.

#### 2.1.2 Transparent glass cover (Fig. 15)

Thickness = 5 mm

**Fig. 15:**
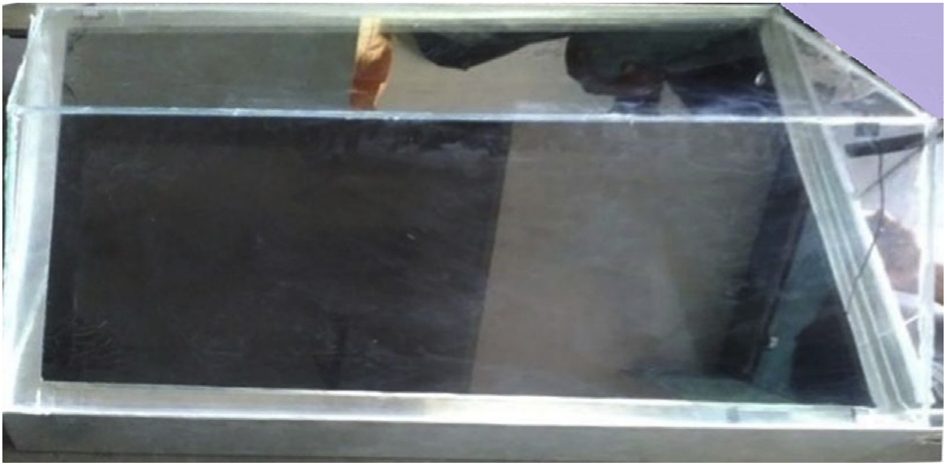
Transparent glass cover.

Area = 720 mm× 900 mm

#### 2.1.3 PCM (Phase Change Material) tank (Fig. 16, Fig. 17 & Table 11)

Area of Tank = 0.9 m × 0.7 m

**Fig. 16:**
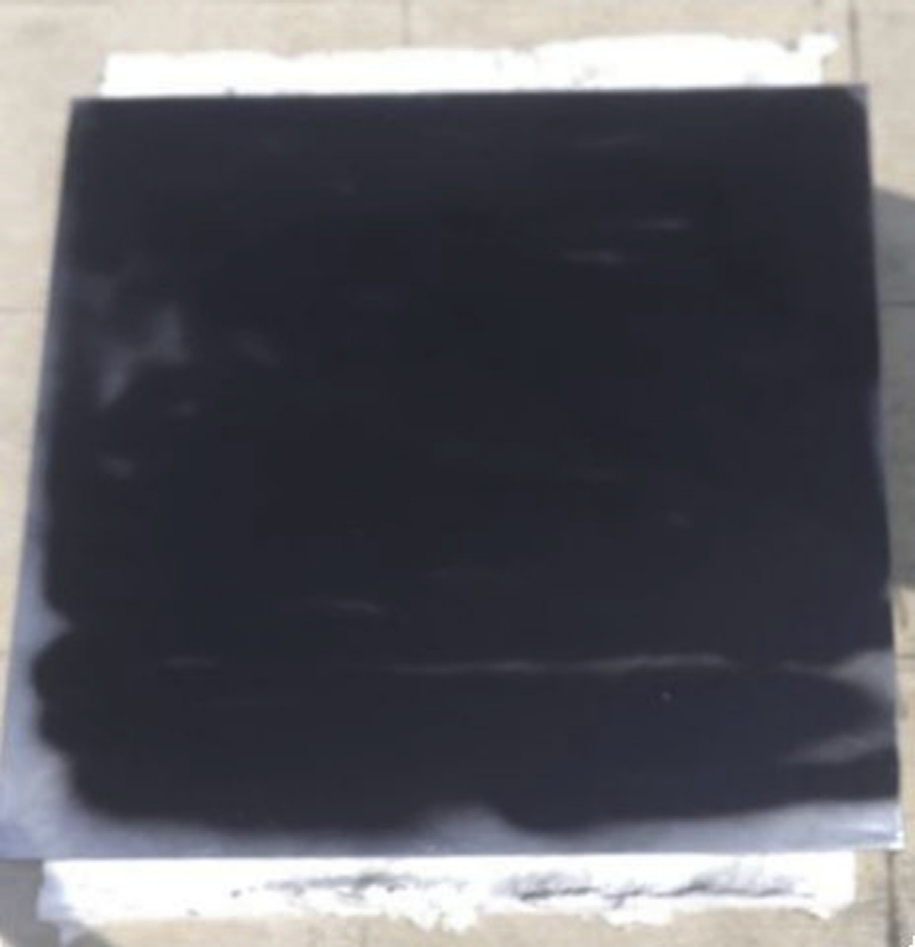
Phase change material (PCM) tank

**Fig. 17:**
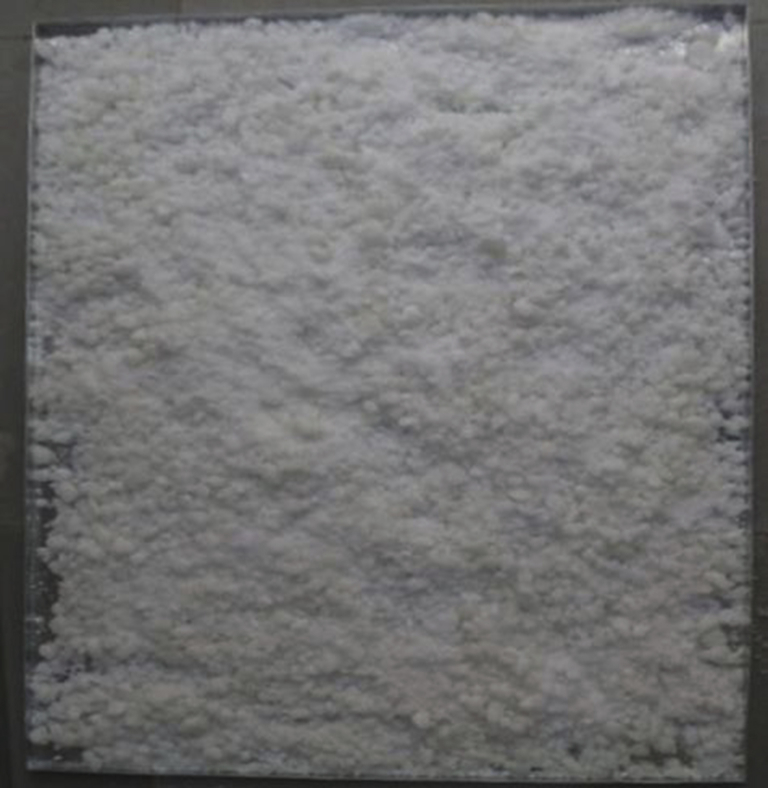
Paraffin wax PCM.

**Table 11:** Properties of paraffin wax – PCM.

Sr. No.	Property	Value
1	Form and appearance	White solid at room temperature
2	Melting temperature	55 °C
3	Latent heat	215 kJ/kg
4	Specific heat	2.14–2.9 kJ/kg *k*

Thickness of Tank = 15 mm

PCM = Paraffin Wax

Quantity = 6 kg

#### 2.1.4 Reflectors

Highly polished silver coated mirrors are used for high reflectivity of ∼0.99; so as to transmit maximum amount of radiation to the basin. Total reflector area is 1.5 m^2^.

#### 2.1.5 Insulation material for basin

Material = Ceramic wool; Thickness = 50 mm

Density = 64kg/m^3^; Thermal Conductivity = 0.25 W/m.K

### 2.2 Efficiency

The efficiency of solar still (Case 1–4)[Table 9]is given by

(1) $$\eta =\;\frac{\sum M_{ew}\times L}{A_{glass}\times I\times t}\times 100$$

The actual maximum efficiency of solar still (Case 5 & 6, using PCM), needs total energy stored in PCM to be taken into account, which is

(2) $$Q={\left\{m\times C_{p}\left(T_{melting}-T_{ambient}\right)\right\}}_{solid}+\left(m\times L_{h}\right)+{\left\{m\times C_{p}\left(T_{max}-T_{melting}\right)\right\}}_{liquid}$$

Therefore, efficiency [Table 9] of solar still using energy storage material is given by

(3) $$\eta =\;\frac{\sum M_{ew}\times L}{\left(A_{glass}\times I\times t\right)+Q}\times 100$$

## Nomenclature

TDS: : Total Dissolved Salts
M_ew_: : Mass of evaporated water
PCM: : Phase Change Material
L: : Latent Heat
RO: : Reverse Osmosis
A_glass_: : Area of glass cover
I: : Incident Radiation Intensity
t: : Time
Q: : Total heat stored
m: : Mass of PCM
C_p_: : Specific heat
T: : Temperature
η: : Efficiency
τ_glass_: : Transmissivity of glass cover
A_reflector_: : Area of Reflector
G.I.: : Galvanized Iron
